# Artifact Correction in Retinal Nerve Fiber Layer Thickness Maps Using Deep Learning and Its Clinical Utility in Glaucoma

**DOI:** 10.1167/tvst.12.11.12

**Published:** 2023-11-07

**Authors:** Min Shi, Jessica A. Sun, Anagha Lokhande, Yu Tian, Yan Luo, Tobias Elze, Lucy Q. Shen, Mengyu Wang

**Affiliations:** 1 Harvard Ophthalmology AI Lab, Schepens Eye Research Institute of Massachusetts Eye and Ear, Harvard Medical School, Boston, MA, USA; 2 Massachusetts Eye and Ear, Harvard Medical School, Boston, MA, USA

**Keywords:** retinal nerve fiber layer thickness, artifact correction, deep learning, visual field prediction, progression forecasting

## Abstract

**Purpose:**

Correcting retinal nerve fiber layer thickness (RNFLT) artifacts in glaucoma with deep learning and evaluate its clinical usefulness.

**Methods:**

We included 24,257 patients with optical coherence tomography and reliable visual field (VF) measurements within 30 days and 3,233 patients with reliable VF series of at least five measurements over ≥4 years. The artifacts are defined as RNFLT less than the known floor value of 50 µm. We selected 27,319 high-quality RNFLT maps with an artifact ratio (AR) of <2% as the ground truth. We created pseudo-artifacts from 21,722 low-quality RNFLT maps with AR of >5% and superimposed them on high-quality RNFLT maps to predict the artifact-free ground truth. We evaluated the impact of artifact correction on the structure–function relationship and progression forecasting.

**Results:**

The mean absolute error and Pearson correlation of the artifact correction were 9.89 µm and 0.90 (*P* < 0.001), respectively. Artifact correction improved R^2^ for VF prediction in RNFLT maps with AR of >10% and AR of >20% up to 0.03 and 0.04 (*P* < 0.001), respectively. Artifact correction improved (*P* < 0.05) the AUC for progression prediction in RNFLT maps with AR of ≤10%, >10%, and >20%: (1) total deviation pointwise progression: 0.68 to 0.69, 0.62 to 0.63, and 0.62 to 0.64; and (2) mean deviation fast progression: 0.67 to 0.68, 0.54 to 0.60, and 0.45 to 0.56.

**Conclusions:**

Artifact correction for RNFLTs improves VF and progression prediction in glaucoma.

**Translational Relevance:**

Our model improves clinical usability of RNFLT maps with artifacts.

## Introduction

Optical coherence tomography (OCT) has been widely used in recent years in the diagnosis and monitoring of glaucoma, which is a progressive optic neuropathy characterized by thinning of the retinal nerve fiber layer (RNFL) and irreversible vision loss.^1^^–^^3^ The circumpapillary RNFL thickness (RNFLT) obtained from spectral domain OCT is the primary structural measurement routinely used for diagnosing and monitoring glaucoma by clinicians.^4^^–^^6^

Although the RNFLT provides critical information on structural damage in glaucoma, imaging artifacts owing to segmentation failures in the context of degraded imaging quality and imaging defects are prevalent, which often compromise the clinical usefulness of the RNFLT. For instance, studies have reported that artifacts were found in 58.5% and 19.9% of circumpapillary RNFL B-scans, 35.4% of optic disc volume RNFL B-scans,^7^^,^^8^ and, more recently, in 43.7% of circumpapillary RNFL B-scans.^9^ These artifacts may compromise the clinical interpretation of the OCT scans for accurate glaucoma diagnosis and progression detection.

Currently, there are no good solutions to correct the RNFLT artifacts, except excluding scans with significant artifacts.^10^ Redoing the OCT scan cannot always eliminate RNFLT artifacts that are due to segmentation failure, because the OCT segmentation algorithm is empirical and imperfect. To address this issue, we have developed a novel deep learning model innovatively enhanced by a contrastive learning module and a consistency learning component to correct RNFLT artifacts. Although we recognize that circumpapillary RNFLT on the 3.46-mm diameter circle is still the dominant RNFLT measurement protocol used by clinicians owing to its dimensional simplicity, more and more studies leveraging machine learning and deep learning for automated glaucoma detection and progression forecasting used the full two-dimensional RNFLT maps to use the more complete structural damage information.^10^^–^^14^ Therefore, in this paper, we focus on correcting artifacts in the entire RNFLT map. The artifacts are defined as RNFLT values less than the known floor value of 50 µm in the literature.^15^^,^^16^ We selected high-quality RNFLT maps with an artifact ratio (AR) of <2% as the ground truth. The AR is defined as the area of artifacts over the area of the RNFLT map excluding the optic disc region. We created pseudo-artifacts from low-quality RNFLT maps with an AR of >5% and superimposed them on high-quality RNFLT maps as model inputs to predict the artifact-free ground truth. We evaluated the artifact correction accuracy by comparing the predicted and original RNFLTs in the region of pseudo-artifacts in the high-quality RNFLT maps. We further evaluated the clinical impact of artifact correction on visual field (VF) prediction and progression forecasting.

## Materials and Methods

This study was approved by the Massachusetts Eye and Ear Institutional Review Board and adhered to the tenets of the Declaration of Helsinki. Given the retrospective nature of the study, the need for informed consent was waived.

### Dataset Description

The dataset used in this study consists of 111,966 RNFLT maps from 24,257 patients tested with available VFs tested within 30 days from the Massachusetts Eye and Ear glaucoma service between 2010 and 2022. Each RNFLT map consisted of 200 × 200 thickness values and was obtained from a spectral domain OCT device (Cirrus, Carl Zeiss Meditec, Dublin, CA). Because this study was designed for artifact correction, we included all OCT scans, regardless of signal strength. We defined artifacts as RNFLT values below the clinically known floor value of 50 µm.^15^^,^^16^ It should be noted that we did not assume RNFLT within the artifact region to be zero. We still used the original RNFLT values in the artifact region during the model training and evaluation. In contrast, we only used reliable 24-2 Humphrey VFs in this study defined as fixation loss of ≤33%, false-positive rate of ≤20%, and false-negative rate of ≤20%. The reliability criteria were also used in our prior studies and are generally applied in the clinical setting.^10^^,^^17^^,^^18^

### Artifact Correction Model

**Figure 1.**
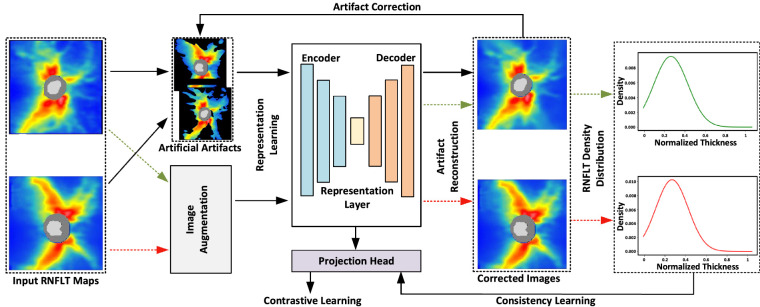
The proposed deep learning framework for artifact correction in RNFLT maps.

We developed a deep learning model RNFLTCorrect (Fig. 1) using a UNet-like architecture to correct RNFLT artifacts.^19^ The deep learning model is composed of three components: (1) an artifact correction backbone^20^; (2) a contrastive learning module to improve artifact correction by enforcing underlying features between an RNFLT map and its perturbated version (e.g., shift and rotation) to be similar^21^; and (3) a consistency learning component to guide the corrected RNFLT maps to have similar statistical distribution with the original RNFLT maps. The deep learning model was optimized by a weighted training target function, which is *L_overall_ = L_correct_ + w_contrast_ × L_contrast_ + w_consistency_ × L_consistency_*. The weight parameters *w_contrast_* and *w_consistency_* scale the impact of contrast loss and consistency loss and were optimized in our model development.

To establish the ground truth, we selected 27,319 high-quality RNFLT maps from 16,669 patients with an AR of <2%. We then created pseudo-artifacts by sampling artifacts from the low-quality RNFLT maps with an AR of >5% and superimposed them onto the high-quality RNFLT maps as model inputs to predict the artifact-free ground truth. The dataset was split into two parts at the patient level, where 70% and 30% of the total data were used for training and testing, respectively. Hence, for each patient , their images are either in the training or testing set, but not both. To evaluate the artifact correction accuracy, we compared the predicted and original RNFLTs in the regions of pseudo-artifacts in the test set. We further assessed the artifact correction accuracy in the artifact region with different ARs including ≤10%, >10%, and >20% for the entire RNFLT map as well as for RNFLTs on the 3.46-mm diameter circle centered on the optic disc.^22^ R^2^ and mean absolute errors (MAEs) were used to measure the artifact correction accuracy.

### Clinical Usefulness Analyses

We conducted two sets of data analyses to demonstrate the clinical usefulness of artifact correction, including VF prediction and progression forecasting.

First, to evaluate the impact of artifact correction on VF prediction, we separately used original and corrected RNFLT maps to predict the accompanying reliable 24-2 VFs tested within 30 days of the OCT scan. For this task, no artifacts were artificially added to the original RNFLT maps. Rather, we used our deep learning model to correct the native artifacts in the original RNFLT maps. Specifically, we trained a VGG-16^23^ model to predict VF mean deviation (MD) and 52 total deviations (TDs) from entire original or artifact-corrected RNFLT maps. R^2^ and MAE were used for assessing prediction accuracy. In addition, we trained a linear regression model to predict VF MD and TDs using RNFLTs on the standard scan circle.

Furthermore, we assessed if artifact correction improves progression and fast progression prediction in glaucoma. We selected original RNFLT maps from patients with at least 5 reliable follow-up VFs across ≥4 years. We used four different progression criteria documented in the literature: (1) MD progression defined as MD slope of <0 and a *P* value of <0.05^24^; (2) TD pointwise regression defined as at least three TD locations with a TD slope of ≤–1 dB/year and a *P* value of <0.05^25^; (3) VF index defined as a VF index slope of <0 and a *P* value of <0.05^26^; (4) MD fast progression defined as an MD slope of ≤–1 dB/year and a *P* value of <0.05.^27^ We trained a VGG-16 model to predict progression from either the original or artifact-corrected RNFLT maps. The area under the receiver operating characteristic curve was used to measure prediction accuracy. In addition, we trained a logistic regression model to predict progression from the RNFLTs on the scan circle.

For VF prediction and progression forecasting tasks, we used the *t*-test combined with three-fold cross-validation. For the three-fold cross-validation, the data were split into three equal subsets, where each subset was iteratively used for model evaluation and the remaining two subsets were combined for model training. The bootstrapping technique was used to compare the prediction accuracy between the models using original and corrected RNFLT maps. All statistical analyses were performed using Python 3.8.

## Results

### Patient and Ophthalmic Characteristics

We included 111,966 OCT scans with VFs tested within 30 days from 42,765 eyes of 24,257 patients in this study. The mean age of participants at the time of imaging was 60.5 ± 16.9 years, and 57.0% were female. Of the 24,257 patients included, 65.4% identified as White (Table 1). Of the 16,543 patients with glaucoma diagnosis information, 71.6% were glaucoma suspects. Of the 42,765 eyes in the dataset, the average RNFLT and vertical cup-to-disc ratio measured by structural OCT were 83.2 ± 5.0 µm and 0.61 ± 0.18, respectively. Intraocular pressure measurements on the same day of the OCT scan were available for 76.5% of the subjects with an average intraocular pressure of 16.0 ± 4.2 mm Hg. Spherical equivalence measurements within 3 months of the OCT scan were available for 43.2% of patients with an average spherical equivalence of –0.9 ± 3.0 diopters. For reliable VFs tested within 30 days of the OCT scan, the MD was –3.5 ± 5.0 dB (Table 1).

**Table 1.** Demographics and Baseline Characteristics of the Patients Included in the Study

Variables	Statistics
No. of patients (eyes)	24,257 (42,765)
No. of RNFLT maps	111,966
Age (years)	60.5 ± 16.9
Gender (female %)	57.0
Race (%) White or Caucasian American Indian or Alaska native Asian Black or African American Native Hawaiian or Other Pacific Islander Unknown or not reported	65.35 0.18 7.58 12.80 0.06 14.03
Intraocular pressure (mm Hg)^a^	16.0 ± 4.2
Spherical equivalent (diopters)^b^	–0.93 ± 3.00
Percentiles of spherical equivalent (diopters) 5 25 50 75 95	−6.13 −1.75 −0.25 0.63 2.50
OCT parameters Average RNFLT (µm) Rim area (mm^2^) Disc area (mm^2^) Average cup-to-disc ratio Cup volume (mm^3^)	83.2 ± 22.4 1.12 ± 0.35 1.94 ± 0.46 0.61 ± 0.18 0.30 ± 0.25
OCT signal strength distribution ≥6 <6	86.5% (8.1 ± 1.1) 13.5% (4.9 ± 1.1)
VF parameter Average HVF MD (dB) Average VFI Average TD (dB)	−3.5 ± 5.0 92.5 ± 14.0 −3.6 ± 6.5
Glaucoma diagnosis (%)^c^ Suspect glaucoma Primary open-angle glaucoma Primary angle-closure glaucoma Other open-angle glaucoma Secondary or unspecified glaucoma subtype	71.6 22.5 1.5 2.5 1.9
Raw artifact distribution ≤10% >10% >20%	No. of RNFLT maps (percentage) 10,0601 (89.8%) 11,365 (10.2%) 4,570 (4.1%)

VFI, visual field index.

All data are presented as mean ± standard deviation, unless otherwise stated.

a Available for 76.5% of patients, measured on the same day as the OCT imaging date.

b Available for 43.2% of patients, with VFs acquired within three months from the OCT imaging date.

c Available for 68.2% of patients.

### OCT Artifact Correction Accuracy

**Figure 2.**
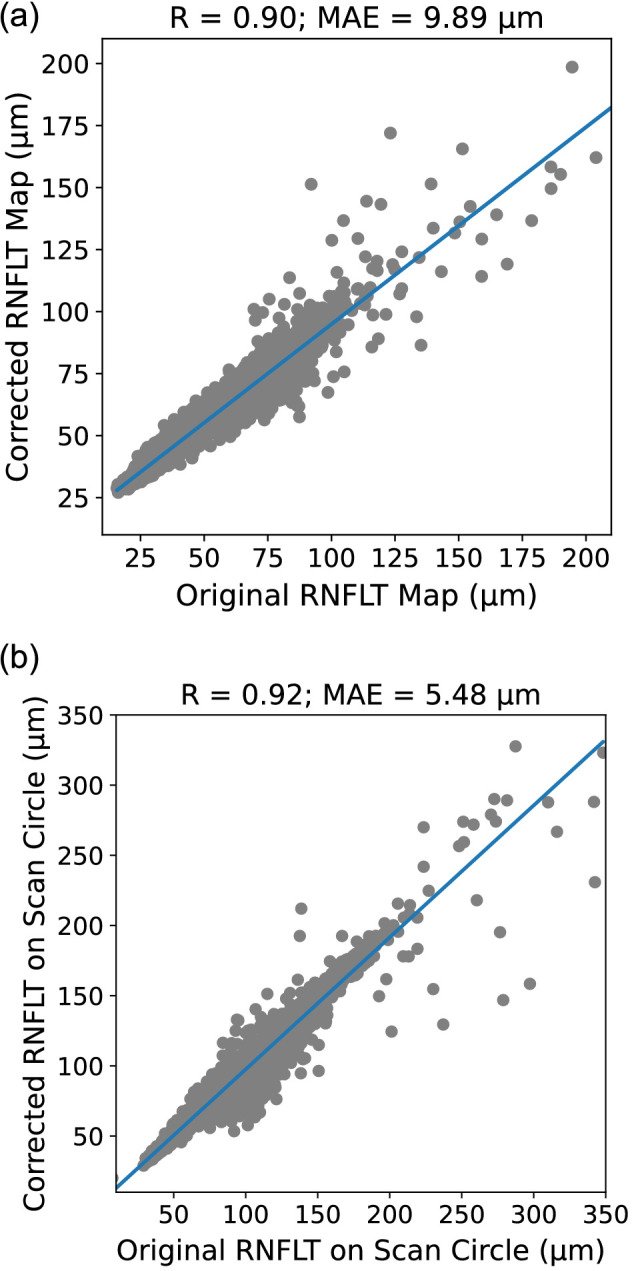
Correlation between original RNFLTs and corrected RNFLT in the artifact region for (a) the entire RNFLT maps and (b) scan circle. MAE, mean average error.

Additional demographic information and ophthalmic characteristics are shown in Supplementary Table S1. Artifact samples from 21,722 low-quality RNFLT maps with an AR of >5% were randomly sampled and added to 27,319 high-quality RNFLT maps with an AR of <2% from 11,798 patients. Of the 27,319 scans with artifacts inserted, 53.4% of scans had an AR of ≤10%, 46.6% of scans had an AR of >10%, and 18.4% of scans had an AR of >20%. Overall, the correction accuracy for artifacts over the entire RNFLT maps achieved an MAE of 9.5 µm; the Pearson correlation (R) between the predicted and true RNFLTs was 0.90 (Fig. 2). The correction accuracy for artifacts on the scan circle was even higher with an MAE of 5.5 µm (R = 0.92; Fig. 2).

When stratified by ARs, artifact correction accuracy in the artifact region decreased with increasing ARs in the RNFLT map. The artifact correction accuracy had MAE of 10.0 µm (R = 0.92) among scans with an AR of ≤10%, MAE of 8.0 µm (R = 0.93) at an AR of >10%, and MAE of 11.1 µm (R = 0.86) at an AR of >20% (Table 2). Similar trends were observed for artifact correction on the scan circle, with MAE of 3.5 µm (R = 0.97) at an AR of ≤10%, MAE of 4.3 µm (R = 0.97) at an AR of >10%, and MAE of 11.9 µm (R = 0.85) at an AR of >20% (Table 2).

**Table 2.** The Artifact Correction Accuracy Measured by MAE and R in the Artifact Region for RNFLT Maps With Overall ARs of ≤10%, >10%, and >20%

AR (No. of RNFLT Maps)		Overall (8,196)	AR ≤ 10% (4,375)	AR > 10% (3,820)	AR > 20% (1,508)
RNFLT maps	R	0.90	0.92	0.89	0.86
	MAE (µm)	9.9	10.0	9.9	11.0
Circle scans	R	0.92	0.97	0.87	0.85
	MAE (µm)	5.5	3.5	10.6	11.9

**Figure 3.**
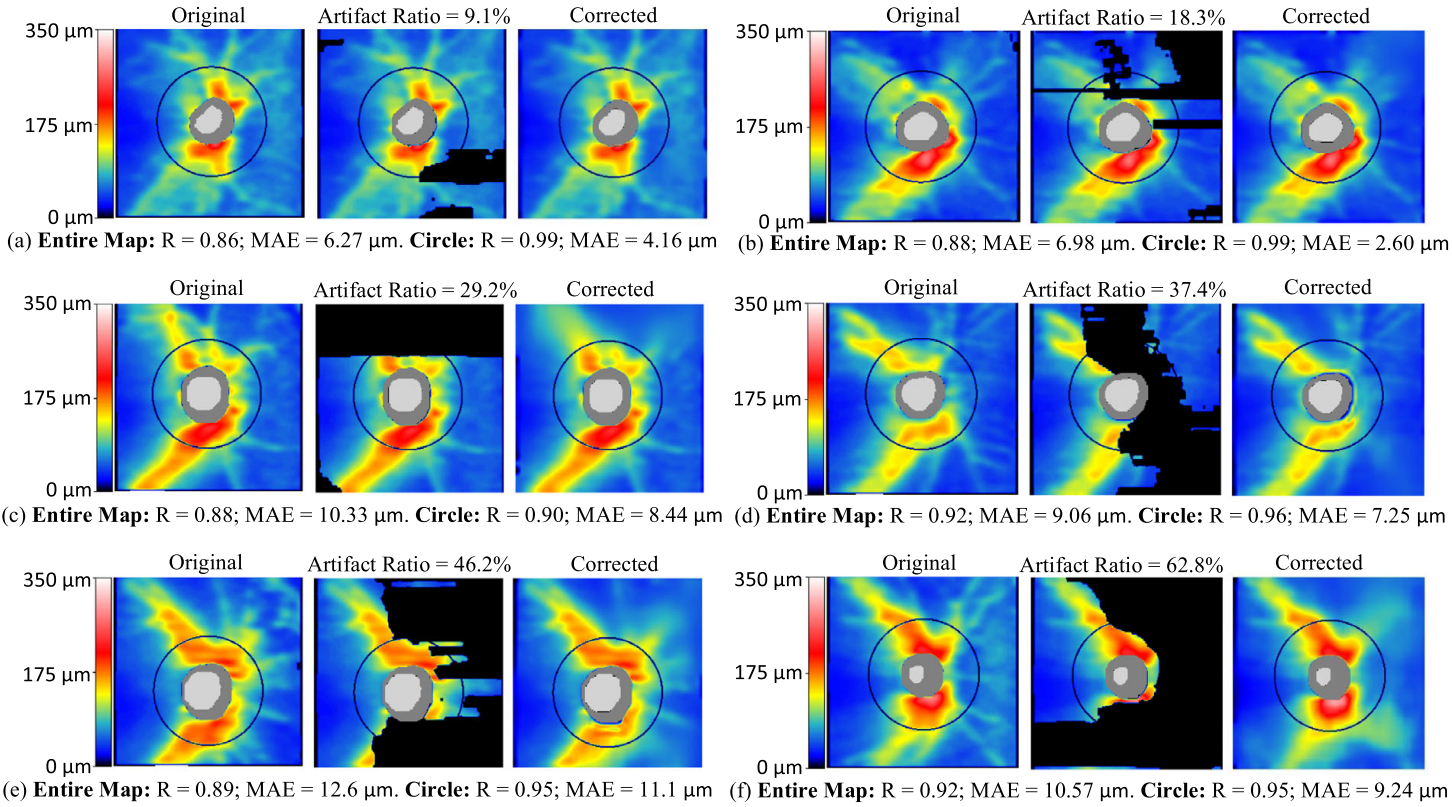
Six examples of RNFLT maps with different ARs ranging from 9.1% to 62.8%. In each example, the left, middle, and right subfigures are the original, artifact-masked, and artifact-corrected RNFLT maps, respectively. Artifact correction accuracy measured by MAE and R for the entire RNFLT map and scan circle are denoted. MAE, mean average error.

Figure 3 shows examples of artifact correction for RNFLT maps with different ARs ranging from 9.1% to 62.8% and correction accuracy in the artifact region measured by R ranging from 0.86 to 0.92 (MAE ranging from 6.3 to 12.6 µm) for the entire RNFLT map and 0.9 to 0.99 for the RNFLTs on the scan circle. Remarkably, as shown in Figures 3e and f, the artifact correction accuracy was still high for RNFLT maps with 46.2% and 62.8% ARs having Rs of 0.89 and 0.92 (MAEs of 12.6 µm and 10.6 µm), respectively. The clinical significance of artifact correction for glaucoma diagnosis was well demonstrated in Figures 3e and f. Clinicians cannot determine if the inferior RNFL bundle is intact or damaged owing to the presence of artifacts. After artifact correction, it is clear that the RNFLT map in Figure 3e had a relatively intact inferior RNFL bundle, while the RNFLT map in Figure 3f had a damaged inferior RNFLT bundle.

### VF Prediction

We used 111,966 pairs of RNFLT maps and VFs tested within 30 days to study the impact of artifact correction on structure–function relationships. Of all RNFLT maps used, 89.8% of the RNFLT maps had AR of ≤10%, 10.2% of the RNFLT maps had an AR of >10%, and 4.1% of the RNFLT maps had an AR of >20% (Table 1).

Overall, MD prediction did not significantly improve with artifact correction (MAE 2.39 dB vs 2.39 dB with correction, *P* = 0.87; R^2^ 0.42 vs R^2^ 0.42 with correction, *P* = 0.28) (Table 3). The MD prediction performance did not significantly differ between using original and corrected RNFLT maps for RNFLT maps with an AR of ≤10%. For RNFLT maps with an AR of >10%, there was mild improvement in R^2^ performance (0.39 without vs 0.40 with correction; *P* < 0.05). For RNFLT maps with an AR of >20%, there was a significant improvement in both MAE and R^2^ after artifact correction (MAE 4.22 dB without vs 4.17 dB with correction, *P* < 0.001; R^2^ 0.32 without vs 0.34 with correction, *P* < 0.001). When using only data from the circle scan, artifact correction demonstrated improvements in MD prediction compared to without correction regardless of ARs (Table 3). MAE improvement after artifact correction notably increased with respect to higher ARs. For RNFLT maps with an AR of >10%, artifact correction improved MAE from 4.3 dB to 4.1 dB (*P* < 0.001) and the R^2^ from 0.10 to 0.17 (*P* < 0.001). For RNFLT maps with an AR of >20%, artifact correction improved MAE from 5.0 dB to 4.7 dB (*P* < 0.001) and the R^2^ from 0.01 to 0.11 (*P* < 0.001).

**Table 3.** MD Predictions Using the Entire RNFLT Map and Scan Circle With Different ARs With and Without Artifact Correction

AR (No. of RNFLT Maps)		Overall (111,966)	AR ≤10% (100,601)	AR >10% (11,365)	AR >20% (4,570)
Raw RNFLT maps	MAE (dB)	2.39	2.27	3.53	4.22
	R^2^	0.42	0.40	0.39	0.32
Corrected RNFLT maps	MAE (dB)	2.39	2.26	3.53	4.17^*^
	R^2^	0.42	0.39	0.40^*^	0.34^*^
Raw circle scans	MAE (dB)	3.07	2.93	4.26	4.99
	R^2^	0.15	0.13	0.10	0.01
Corrected circle scans	MAE (dB)	3.02^*^	2.89^*^	4.13^*^	4.74^*^
	R^2^	0.20^*^	0.17^*^	0.17^*^	0.11^*^

R^2^, coefficient of determination.

* *P* < 0.05.

**Figure 4.**
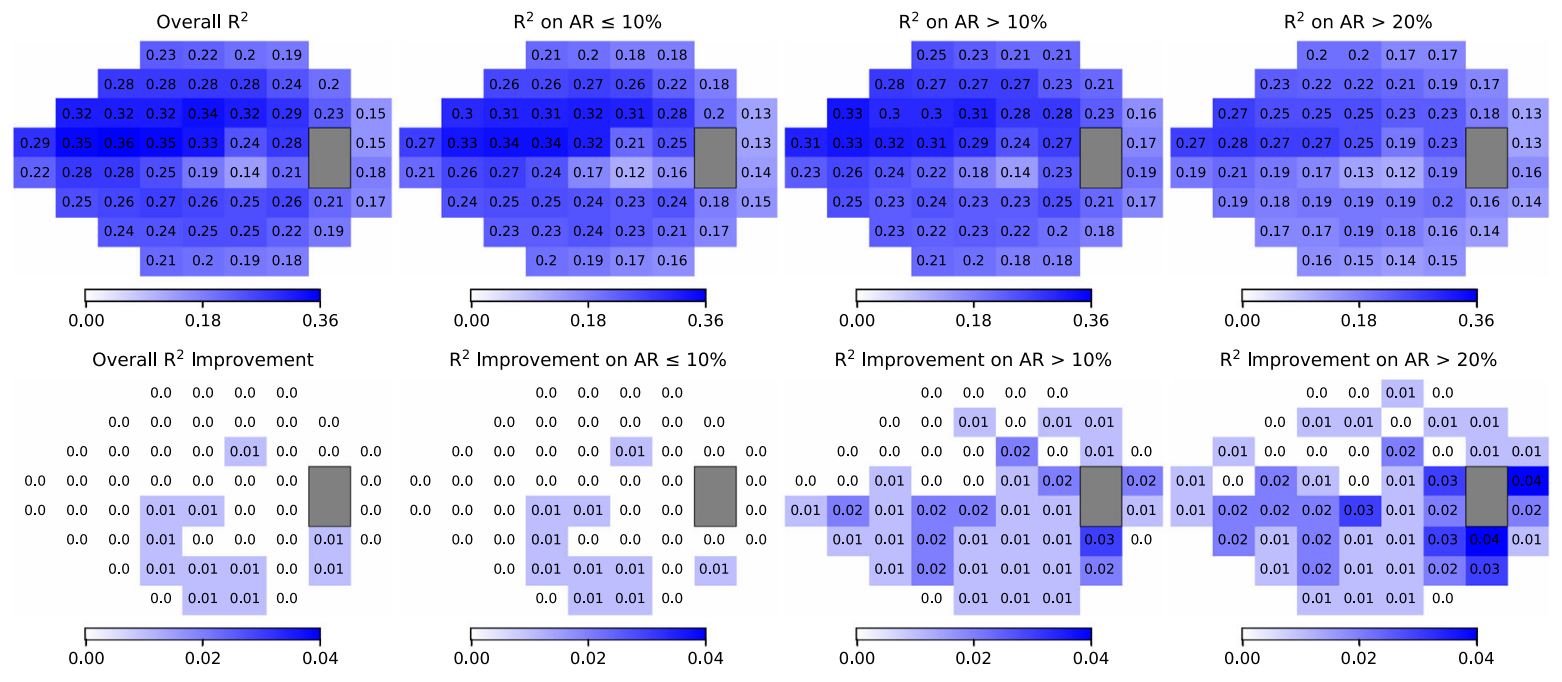
The first row shows the R2 performance of VF TD prediction using the original RNFLT map. The second row shows the R2 performance improvement of TD prediction using the artifact-corrected RNFLT map compared with the original RNFLT map. The four columns from left to right are results for all RNFLT maps, and RNFLT maps with an AR of ≤10%, an AR of >10%, and an AR of >20%, respectively. Zero indicates no significant improvement for the respective VF location (*P* < 0.05 is considered as significant). R^2^, coefficient of determination.

Figure 4 shows R^2^ performance on predicting 52 TD values using original RNFLT maps versus corrected RNFLT maps. Overall, artifact correction significantly improved TD prediction for 11 VF locations with a ≤0.01 R^2^ improvement, which were mainly in the inferior hemifield. When stratified by ARs, the improvement in R^2^ with artifact correction increased with increasing ARs (Fig. 4). For RNFLT maps with an AR of >10% and an AR of >20%, 36 and 41 VF locations had improved R^2^ of ≤0.02 and 0.03, respectively, whereas for RNFLT maps with an AR of ≤10%, the R^2^ improvement was more modest (≤0.01) with 10 locations improved. For the MAE performance improvement, see Supplementary Figure S1. See TD predictions using the RNFLTs on the scan circle in Supplementary Figures S2 and S3.

### Glaucoma Progression Prediction

We identified 19,070 RNFLT maps from 5436 eyes of 3233 patients with 6.6 ± 1.8 reliable VFs within 6.8 ± 1.7 years of follow-up. The demographic and ophthalmic characteristics are summarized in Supplementary Table S1. Of all raw RNFLT maps with native artifacts, 86.8% had an AR of ≤10%, 13.2% had an AR of >10%, and 5.4% had an AR of >20% (Supplementary Table S1). Artifact correction generally improved (*P* < 0.05) the areas under the receiver operating characteristic curve for progression forecasting in RNFLT maps with an AR of ≤10%, >10%, and >20% (Table 4): (1) MD progression: 0.64 to 0.65, 0.556 to 0.559, and 0.51 to 0.52; (2) MD fast progression: 0.67 to 0.68, 0.54 to 0.60, and 0.45 to 0.56; (3) and TD pointwise progression: 0.68 to 0.69, 0.62 to 0.63, and 0.62 to 0.64. For VF index progression, artifact correction only improved the progression prediction for RNFLT maps with an AR of >10% and an AR of >20% from 0.57 to 0.59 and 0.53 to 0.56, respectively. Progression prediction only using RNFLTs on the scan circle is shown in Supplementary Table S2.

**Table 4.** Glaucoma Progression Forecasting Using the RNFLT Maps With Different ARs With and Without Artifact Correction

Progression Type	AR (No. of Eyes)	Overall (19,070)	AR ≤10% (16,559)	AR > 10% (2,511)	AR > 20% (1,023)
MD	Raw RNFLT map	0.63	0.64	0.556	0.51
	Corrected RNFLT map	0.64^*^	0.65^*^	0.559^*^	0.52^*^
VFI	Raw RNFLT map	0.630	0.636	0.57	0.53
	Corrected RNFLT map	0.627	0.633	0.59^*^	0.56^*^
TD pointwise	Raw RNFLT map	0.68	0.68	0.62	0.62
	Corrected RNFLT map	0.69^*^	0.69^*^	0.63^*^	0.64^*^
MD fast	Raw RNFLT map	0.65	0.67	0.54	0.45
	Corrected RNFLT map	0.67^*^	0.68^*^	0.60^*^	0.56^*^

AUC, area under the receiver operating characteristic curve; VFI, visual field index.

* *P* < 0.05.

## Discussion

To the best of our knowledge, this research is the first work in the literature to correct RNFLT artifacts with deep learning. RNFLT measurement is an important tool for glaucoma diagnosis and monitoring. We demonstrated that our artifact correction model RNFLTCorrect was able to predict the true RNFLTs in the artifact region with high accuracy, evidenced by correlations of 0.90 and 0.92 for the artifact region over the entire map and the scan circle, respectively (Table 2). We have shown that using deep learning to correct artifacts improved structure–function relationships in the context of using RNFLT maps to predict VFs with deep learning. More important, artifact correction can significantly improve progression prediction in RNFLT maps with an AR of >10% and an AR of >20%. Although the improvement for structure–function relationship and progression forecasting is not substantial, it is beneficial for clinical studies to remove and correct RNFLT artifacts by simply implementing our model.

High-quality OCT imaging is critical in clinical care for glaucoma. Imaging artifacts can impede effective clinical interpretation and progression detection, especially when the artifact is large so that the structural measurement is therefore not assessable by clinicians.^9^^,^^28^^,^^29^ Prior efforts addressing artifacts in OCT imaging have primarily focused on denoising the cross-sectional B-scans.^30^^–^^32^ These models use deep neural networks to reduce speckle noise in B-scans and improve the overall image quality. However, the postprocessed RNFLT is the primary structural measurement routinely used by glaucoma specialists, as opposed to B-scans. The RNFLT artifacts are due to segmentation failure largely influenced by degraded imaging quality and imaging defects. Even if the imaging quality measured by signal strength is high, RNFLT artifacts owing to segmentation failure can still occur quite often. Our model uniquely addresses such imaging artifacts with a deep learning approach. The model can also be easily extended to correct artifacts for other ophthalmic imaging modalities, such as fundus images and macular scans, to benefit the broader clinical and research communities.

Using deep learning to assess structure–function associations has been extensively studied in prior work.^12^^,^^33^^–^^35^ These models generally use peripapillary RNFLT maps to forecast VF defects and have demonstrated impressive accuracy.^12^^,^^36^ However, existing deep learning methods have not addressed the potential negative impact of RNFLT artifacts on VF prediction. In this paper, our results have shown that artifact correction can generally improve the VF prediction, especially for RNFLT maps with higher ARs (e.g., an AR of >20%).

More important, prior works have attempted to predict glaucoma progression by deep learning.^37^^–^^39^ None of the prior works have addressed the issue of RNFLT artifacts. In our work, we demonstrated that artifact correction can substantially improve the prediction of progression (0.02 area under the receiver operating characteristic curve improvement for TD pointwise progression) and fast progression (0.11 for MD fast progression), especially in RNFLT maps with an AR of >20%. Although OCT images with an AR of >20% only make up a small proportion of our dataset (4.1%), more accurate prediction of the progression of an irreversible blinding disease such as glaucoma can have a significant impact on the patient's quality of life. Given that OCT imaging and VF testing are performed annually in most glaucoma care settings, our algorithm potentially can alert clinicians to repeat testing sooner to confirm progression and provide effective treatment.

There are some limitations to this study. First, we selected high-quality RNFLT maps to train the RNFLTCorrect model, but these maps may also contain minor artifacts around the peripheral regions, which are not perfect ground truth for training our artifact correction model.^40^ Second, although we evaluated the advantage of artifact correction with VF prediction and progression forecasting in glaucoma using original RNFLT maps with native artifacts from 24,257 glaucoma patients, most of the RNFLT maps had an AR of ≤10% (Table 1). It is possible that we had a disproportionate number of maps with very few artifacts to correct, which may explain the lack of significant improvement in the overall TD and MD prediction (Table 3 and Fig. 4). Third, although we have demonstrated the benefits of artifact correction for RNFLT maps from Cirrus OCT, we have yet to validate this model on other manufacturer devices, such as Topcon or Heidelberg, or on other imaging modalities such as fundus images and macular scans. Finally, we identified artifacts that caused RNFLT to be less than the known floor value of 50 µm as a result of segmentation failure. Various factors can cause such segmentation failure, including blocked imaging signal owing to vitreous opacity and retinal layer disruption such as epiretinal membrane owing to retinal diseases such as diabetic retinopathy and age-related macular degeneration. Our model is not able to distinguish the source of segmentation failure.

The performance in VF prediction and glaucoma progression forecasting typically improves with a reduced AR. This result is attributed to the availability of more RNFLT information when the AR is smaller. Conversely, the enhancements in VF prediction and progression forecasting are more pronounced with a higher AR, as seen in Tables 3, 4, and Figure 4. This phenomenon arises because, with a larger AR, the RNFLT or circle scan often loses a greater portion of its thickness information. The absence of crucial thickness data, particularly in the RNFLT bundles, hampers the capability of the RNFLT or circle scan in predicting VF and glaucoma progression. However, after artifact correction, the lost thickness information is restored, leading to a marked improvement in performance compared with the original RNFLT map or circle scan.

In conclusion, the prevalence of artifacts in a large proportion of RNFLT maps remains a significant clinical challenge for effectively using OCT measurements to diagnose and prognose glaucoma. We have developed a novel deep learning framework termed RNFLTCorrect to address OCT artifacts, consisting of an artifact correction backbone together with a contrastive learning module and a consistency learning component. RNFLTCorrect has achieved high accuracy on artifact correction, which improves the VF prediction and progression forecasting in glaucoma.

## Supplementary Material

Supplement 1

Supplement 2
