# Supplementary material for: Artifact Correction in Retinal Nerve Fiber Layer Thickness Maps Using Deep Learning and Its Clinical Utility in Glaucoma
Source: Transl Vis Sci Technol. 2023 Nov 7;12(11):12. doi: 10.1167/tvst.12.11.12 (PMC10631515; doi:10.1167/tvst.12.11.12)
Supplement: Supplement 1 [file tvst-12-11-12_s001.pdf]

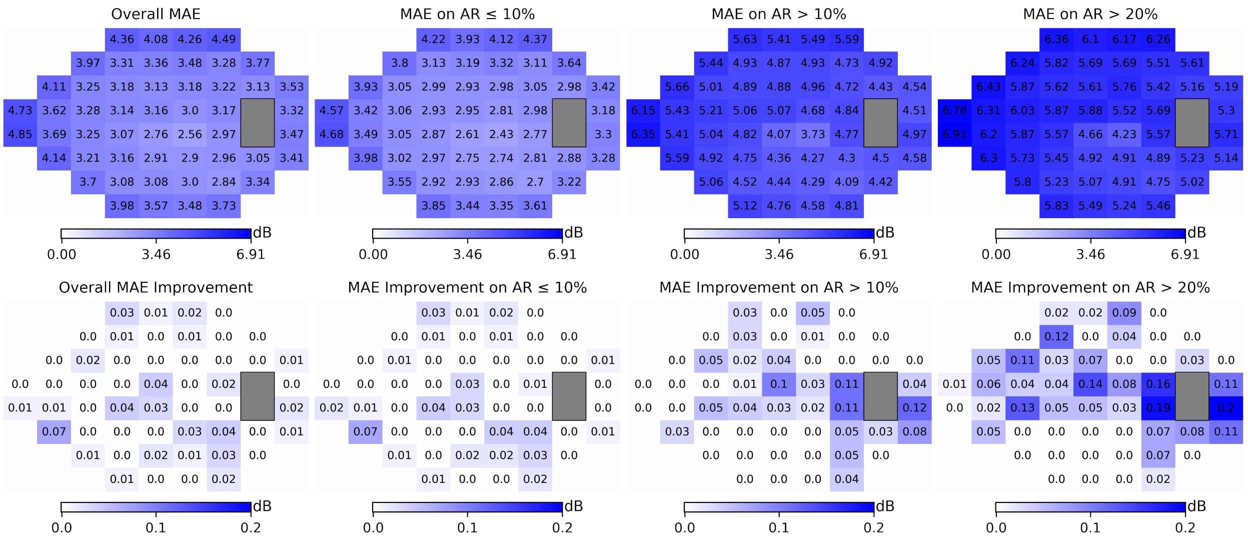

Supplementary Figure 1 The first row shows the MAE performance of VF TD prediction using the original RNFLT map. The second row shows the MAE performance improvement of TD prediction using the artifact-corrected RNFLT map compared with the original RNFLT map. The four columns from left to right are results for all RNFLT maps, and RNFLT maps with AR ≤ 10%, AR > 10%, AR > 20%, respectively. Zero indicates no significant improvement for the respective visual field location ($p < 0.05$ is considered as significant). VF: visual field; TD: total deviation; MAE: mean absolute error; RNFLT: retinal nerve fiber layer thickness; AR: artifact ratio.

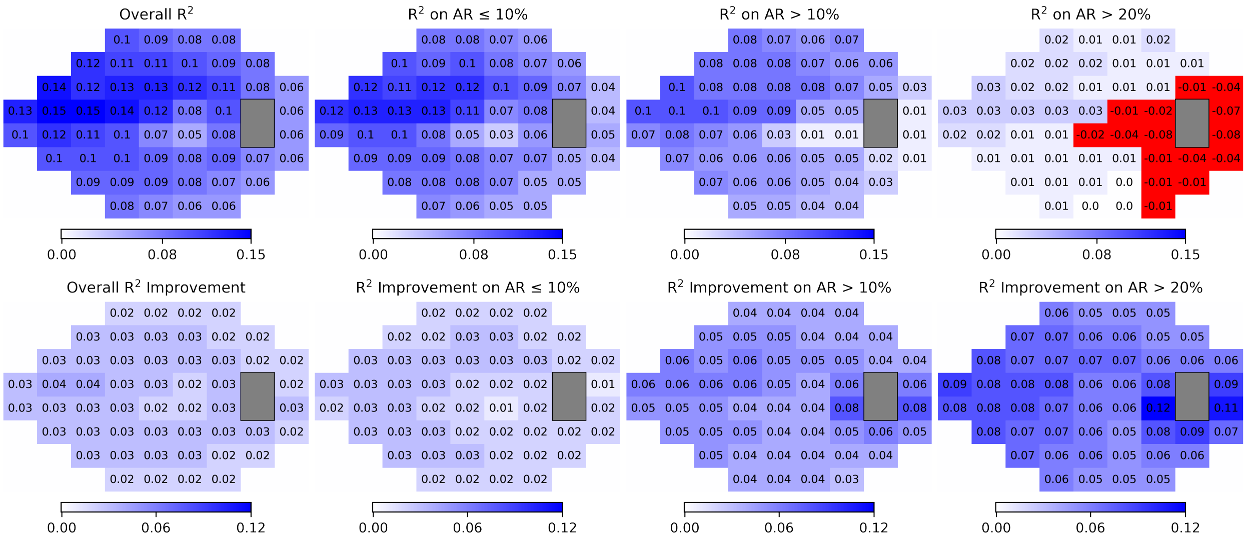

Supplementary Figure 2 The first row shows the R^2 performance of VF TD prediction using RNFLTs from the scan circle. The second row shows the R^2 performance improvement of TD prediction using the artifact-corrected RNFLT map compared with the original RNFLT map. The four columns from left to right are results for all RNFLT maps, and RNFLT maps with AR ≤ 10%, AR > 10%, AR > 20%, respectively. Zero indicates no significant improvement for the respective visual field location ($p < 0.05$ is considered as significant). VF: visual field; TD: total deviation; R^2, coefficient of determination; RNFLT: retinal nerve fiber layer thickness; AR: artifact ratio.

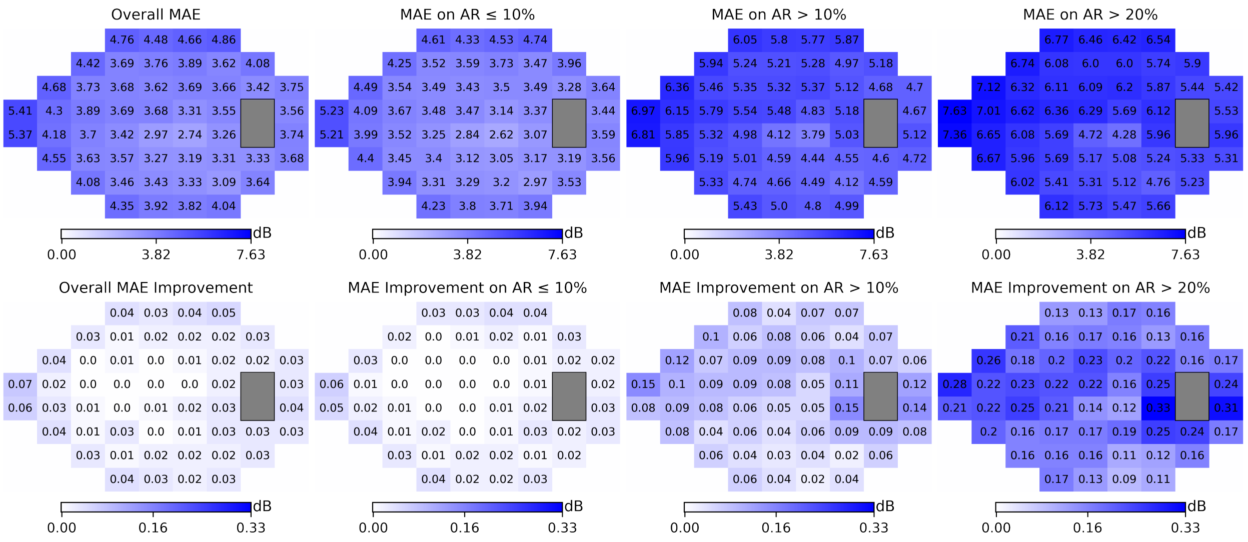

Supplementary Figure 3 The first row shows the MAE performance of VF TD prediction using RNFLTs from the scan circle. The second row shows the MAE performance improvement of TD prediction using the artifact-corrected RNFLT map compared with the original RNFLT map. The four columns from left to right are results for all RNFLT maps, and RNFLT maps with AR ≤ 10%, AR > 10%, AR > 20%, respectively. Zero indicates no significant improvement for the respective visual field location ($p < 0.05$ is considered as significant). VF: visual field; TD: total deviation; MAE: mean absolute error; RNFLT: retinal nerve fiber layer thickness; AR: artifact ratio.
