# Supplementary material for: Artifact Correction in Retinal Nerve Fiber Layer Thickness Maps Using Deep Learning and Its Clinical Utility in Glaucoma
Source: Transl Vis Sci Technol. 2023 Nov 7;12(11):12. doi: 10.1167/tvst.12.11.12 (PMC10631515; doi:10.1167/tvst.12.11.12)
Supplement: Supplement 2 [file tvst-12-11-12_s002.pdf]

Supplemental Table 1: Demographics and baseline characteristics of the patients included in three tasks including artifact correction model development, VF prediction, and progression forecasting. OCT: optical coherence tomography; RNFL: retinal nerve fiber layer; RNFLT: RNFL thickness; VF: visual field; MD: mean deviation; VFI: visual field index; TD: total deviation. All data are presented as mean ± standard deviation unless otherwise stated.

**Selected high-quality data for developing and testing our artifact correction model.**	
**Variables**	**Statistics**
Number of patients (eyes)	11,798 (16,669)
Number of RNFLT maps	27,319
Age (years)	56.9 ± 16.6
Gender (female %)	60.0
Race (%)	
• White or Caucasian	63.92
• American Indian or Alaska native	0.18
• Asian	8.30
• Black or African American	12.84
• Native Hawaiian or Other Pacific Islander	0.05
• Unknown or not reported	14.71
OCT parameters	
• Average RNFL thickness (μm)	94.1 ± 19.1
• Rim area (mm^2)	1.2 ± 0.3
• Disc area (mm^2)	2.0 ± 0.4
• Average cup to disc ratio	0.6 ± 0.2
• Cup volume (mm^3)	0.3 ± 0.2
OCT signal strength distribution	
• signal strength ≥ 6	95.6% (8.4 ± 1.0)
• signal strength < 6	4.4% (5.4 ± 0.6)
Visual field parameter	
• Average HVF MD (dB)	-1.8 ± 2.3
• Average VFI	96.8 ± 7.1
• Average TD (dB)	-1.8 ± 4.2
Artificially generated artifact distribution	number of RNFLT maps (percentage)
• Artifact ratio ≤ 10%	14,584 (53.4%)
• Artifact ratio > 10%	12,735 (46.6%)
• Artifact ratio > 20%	5,026 (18.4%)
**Cross-sectional data used to evaluate the visual function prediction in glaucoma**	
**Variables**	**Statistics**
Number of patients (eyes)	24,257 (42,765)
Number of RNFLT maps	111,966
Age (years)	60.5 ± 16.9

Gender (female %)	57.0
Race (%)<br>• White or Caucasian<br>• American Indian or Alaska native<br>• Asian<br>• Black or African American<br>• Native Hawaiian or Other Pacific Islander<br>• Unknown or not reported	 65.35<br>0.18<br>7.58<br>12.80<br>0.06<br>14.03
OCT parameters<br>• Average RNFL thickness (μm)<br>• Rim area (mm^2)<br>• Disc area (mm^2)<br>• Average cup to disc ratio<br>• Cup volume (mm^3)	 83.2 ± 22.4<br>1.1 ± 0.4<br>1.9 ± 0.5<br>0.6 ± 0.2<br>0.30 ± 0.3
OCT signal strength distribution<br>• signal strength ≥ 6<br>• signal strength < 6	 86.5% (8.1 ± 1.1)<br>13.5% (4.9 ± 1.1)
Visual field parameter<br>• Average HVF MD (dB)<br>• Average VFI<br>• Average TD (dB)	 -3.5 ± 5.0<br>92.5 ± 14.0<br>-3.6 ± 6.5
Native artifact distribution<br>• Artifact ratio ≤ 10%<br>• Artifact ratio > 10%<br>• Artifact ratio > 20%	number of RNFLT maps (percentage)<br>10,0601 (89.7%)<br>11,365 (10.2%)<br>4,570 (4.1%)
**Longitudinal data for progression forecasting**	
**Variables**	**Statistics**
Number of patients (eyes)	3,233 (5,436)
Number of RNFLT maps	19,070
Age (years)	63.3 ± 12.3
Gender (female %)	55.3
Race (%)<br>• White or Caucasian<br>• American Indian or Alaska native<br>• Asian<br>• Black or African American<br>• Native Hawaiian or Other Pacific Islander<br>• Unknown or not reported	 70.56<br>0.12<br>8.57<br>13.24<br>0.03<br>7.48
OCT parameters<br>• Average RNFL thickness (μm)<br>• Rim area (mm^2)<br>• Disc area (mm^2)<br>• Average cup to disc ratio<br>• Cup volume (mm^3)	 80.1 ± 18.0<br>1.1 ± 0.3<br>1.9 ± 0.5<br>0.6 ± 0.2<br>0.3 ± 0.3
OCT signal strength distribution<br>• signal strength ≥ 6<br>• signal strength < 6	 86.2% (8.0 ± 1.0)<br>13.8% (5.0 ± 1.0)
Visual field parameter (dB)<br>• Average HVF MD<br>• Average VFI<br>• Average TD	 -3.2 ± 3.9<br>93.8 ± 10.3<br>-3.3 ± 5.6

• Average number of measurements<br>• Average number of years of follow-up	5.9 ± 4.9<br>1.7 ± 1.7
Progression outcome prevalence (%)<br>• MD<br>• MD Fast<br>• VFI<br>• TD Pointwise	 9.8<br>2.7<br>11.5<br>12.0
Native artifact distribution<br>• Artifact ratio ≤ 10%<br>• Artifact ratio > 10%<br>• Artifact ratio > 20%	number of RNFLT maps (percentage)<br>16,559 (86.8%)<br>2,511 (13.2%)<br>1,023 (5.4%)

Supplemental Table 2: Glaucoma progression forecasting using the RNFLTs on the scan circle with different ARs with and without artifact correction. Statistically significant results ($p < 0.05$) are marked with asterisks. RNFLT: retinal nerve fiber layer thickness; VF: visual field; AUC: area under the receiver operating characteristic curve; AR: artifact ratio; MD: mean deviation; VFI: visual field index; TD: total deviation.

Progression type	Artifact Ratio (Number of eyes)	Overall (19,070)	AR ≤10% (16,559)	AR >10% (2,511)	AR >20% (1,023)
MD	Raw Circle Scan	0.617	0.629	0.53	0.46
	Corrected Circle Scan	0.620*	0.629	0.55*	0.49*
VFI	Raw Circle Scan	0.624	0.632	0.57	0.53
	Corrected Circle Scan	0.625	0.631	0.59*	0.56*
TD Pointwise	Raw Circle Scan	0.681	0.684	0.62	0.60
	Corrected Circle Scan	0.686*	0.685	0.65*	0.64*
MD Fast	Raw Circle Scan	0.627	0.631	0.56	0.50
	Corrected Circle Scan	0.632*	0.633	0.58*	0.52*
